# Anti-CD40 Antibody Fused to CD40 Ligand Is a Superagonist Platform for Adjuvant Intrinsic DC-Targeting Vaccines

**DOI:** 10.3389/fimmu.2021.786144

**Published:** 2022-01-13

**Authors:** Valentina Ceglia, Sandra Zurawski, Monica Montes, Mitchell Kroll, Aurélie Bouteau, Zhiqing Wang, Jerome Ellis, Botond Z. Igyártó, Yves Lévy, Gerard Zurawski

**Affiliations:** ^1^ Baylor Scott and White Research Institute, Dallas, TX, United States; ^2^ Université Paris-Est Créteil, Sciences de la Vie et de la Santé, Créteil, France; ^3^ Vaccine Research Institute, The Institut National de la Santé et de la Recherche Médicale (INSERM), Unité U955, Institut Mondor de Recherche Biomédicale, Créteil, France; ^4^ Institute of Biomedical Studies, Baylor University, Waco, TX, United States; ^5^ Department of Microbiology and Immunology, Thomas Jefferson University, Philadelphia, PA, United States

**Keywords:** dendritic cells, monoclonal antibodies, superagonist, adjuvant-intrinsic, vaccine

## Abstract

CD40 is a potent activating receptor expressed on antigen-presenting cells (APCs) of the immune system. CD40 regulates many aspects of B and T cell immunity *via* interaction with CD40L expressed on activated T cells. Targeting antigens to CD40 *via* agonistic anti-CD40 antibody fusions promotes both humoral and cellular immunity, but current anti-CD40 antibody-antigen vaccine prototypes require co-adjuvant administration for significant *in vivo* efficacy. This may be a consequence of dulling of anti-CD40 agonist activity *via* antigen fusion. We previously demonstrated that direct fusion of CD40L to anti-CD40 antibodies confers superagonist properties. Here we show that anti-CD40-CD40L-antigen fusion constructs retain strong agonist activity, particularly for activation of dendritic cells (DCs). Therefore, we tested anti-CD40-CD40L antibody fused to antigens for eliciting immune responses *in vitro* and *in vivo*. In PBMC cultures from HIV-1-infected donors, anti-CD40-CD40L fused to HIV-1 antigens preferentially expanded HIV-1-specific CD8^+^ T cells *versus* CD4^+^ T cells compared to analogous anti-CD40-antigen constructs. In normal donors, anti-CD40-CD40L-mediated delivery of Influenza M1 protein elicited M1-specific T cell expansion at lower doses compared to anti-CD40-mediated delivery. Also, on human myeloid-derived dendritic cells, anti-CD40-CD40L-melanoma gp100 peptide induced more sustained Class I antigen presentation compared to anti-CD40-gp100 peptide. In human CD40 transgenic mice, anti-CD40-CD40L-HIV-1 gp140 administered without adjuvant elicited superior antibody responses compared to anti-CD40-gp140 antigen without fused CD40L. In human CD40 mice, compared to the anti-CD40 vehicle, anti-CD40-CD40L delivery of Eα 52-68 peptide elicited proliferating of TCR I-Eα 52-68 CD4^+^ T cells producing cytokine IFNγ. Also, compared to controls, only anti-CD40-CD40L-Cyclin D1 vaccination of human CD40 mice reduced implanted EO771.LMB breast tumor cell growth. These data demonstrate that human CD40-CD40L antibody fused to antigens maintains highly agonistic activity and generates immune responses distinct from existing low agonist anti-CD40 targeting formats. These advantages were *in vitro* skewing responses towards CD8^+^ T cells, increased efficacy at low doses, and longevity of MHC Class I peptide display; and in mouse models, a more robust humoral response, more activated CD4^+^ T cells, and control of tumor growth. Thus, the anti-CD40-CD40L format offers an alternate DC-targeting platform with unique properties, including intrinsic adjuvant activity.

## Introduction

CD40 is a potent activating TNFR superfamily member expressed on antigen-presenting cells (APCs) (1). Agonistic anti-CD40 monoclonal antibodies (mAbs) are in clinical development based on the notion of directly activating APCs to stimulate immune responses either against intrinsic antigens, e.g., tumor-associated antigens (TAAs), or as an adjuvant to protein or peptide vaccines (2–4). Directly linking antigens to anti-CD40 antibodies by chemical conjugation (5), non-covalent assembly (6), or direct fusion (7) elicits potent antigen-specific cellular and humoral immunity at very low antigen doses in a wide array of *in vitro* and *in vivo* settings (8–11). In particular, antigen-targeting to CD40 elicits superior cellular T cell responses compared to targeting other specialized antigen-presenting Dendritic Cell (DC) receptors, likely due to antigen accumulation within the early endosome compartment, as distinct to the rapid antigen entry into late endosomes characterized by targeting other DC receptors (8, 12).

Potent activation of CD40 is not required for efficient Class I and Class II presentation of antigens *via* CD40-targeting *in vitro* (7, 12); however, *in vivo* efficacy requires co-administration of Toll-like receptor (TLR) activating agents such as poly IC (8–11). Nonetheless, these *in vitro* and *in vivo* studies utilized anti-CD40 antibody-antigen complexes or fusions with low CD40 agonist activity (7, 10), and the clear benefit of agonistic anti-CD40 antibody combined with poly IC for peptide-based vaccination in non-human primates (3) suggests CD40-targeting of antigens may be further improved by utilizing fully agonistic anti-CD40 targeting vehicles.

In this study, we demonstrate that previously described superagonist forms of anti-CD40 antibody generated by fusion to CD40L (13) retain strong agonist activity when also linked to various antigens. Our studies show that anti-CD40-CD40L-antigen constructs alter the nature of expanded antigen-specific memory T cells *in vitro*, and *in vivo* can promote significant cellular and humoral responses without co-administered adjuvant, and thus have potential as adjuvant-intrinsic DC-targeting vaccine vehicles.

## Material and Methods

### Anti-Human CD40 Monoclonal Antibodies and Antigen Adducts

Sequences and production methods for in-house-derived 12E12, 11B6, 24A3, 12B4, and CP [CP-870,893, a Pfizer Inc. agonistic antibody (4)] anti-human CD40 human IgG4 antibodies have been described (13). The HIV-5pep antigen cassette (Flex-v1-Pep-gag17-f1-gag253-f2-nef116-f3-nef66-f4-pol158) attached to H chain C-termini has been detailed (7, 14) and is contained within GenPept Sequence ID: AJD85777.1. The HPV16.E6/7 H chain adduct (9) has been described (GenBank KP684039). The Dockerin V1 (Doc) H chain adducts and Cohesin-Flu M1 protein were described elsewhere (6). Other antigen adducts are described in the sections relevant to particular protocols.

### B Cell Proliferation and DC Activation

B cell proliferation assays and DC activation assays were performed as previously described (13). Briefly, B cell assays used CFSE-labeled human peripheral blood mononuclear cells (PBMCs) incubated with a dose range of the test articles with human IL-4 and human IL-21 for 5 days of culture, then cells were stained with surface and live-dead markers followed by flow cytometry analysis gating on single live cells and CFSE^−^/CD19^+^ cells. To account for donor variation, independent experiments were often collated by normalizing to the baseline and maximal responses in each experiment. Typically, baseline percentages across independent experiments using different donors were consistently in the range of ~1 to ~10% for the antibodies administered alone, and ~4 to ~22% for the antibodies administered with the sCD40L. Maximum proliferation among the experiments typically was in the range of ~46 to 97%. For DC activation, myeloid-derived human dendritic cells (MDDC), from human blood monocytes cultured with human IL-4 and human GM-CSF for 5 days, were incubated with IL-4 and GM-CSF and a dose range of the test articles. After 24 or 48 h, supernatants were analyzed for secreted cytokines by Luminex^®^, and in some cases the cells were stained for cell surface activation markers.

### Surface Plasmon Resonance

SPR analyses were performed as previously described (13).

### T Cell Expansion Assay

Cryopreserved human PBMC were thawed with 50 U/ml benzonase^®^ nuclease (Millipore, cat 70746), washed and rested overnight in RPMI 1640 enriched with 100X PenStrep (Gibco, 15140-122), 100X Hepes Buffer (Gibco, 15630-080), 100X Non-essential amino acids (NEAA) (Gibco, 11140-050), 100X Sodium Pyruvate (Gibco, 11360-070), 1000X 2-Mercaptoethanol (Gibco, 21985- 023), 100X Glutamax (Gibco, 35050-61) (herein called complete RPMI 1640) with 10% AB serum (GemCell, 100-512) in a 37°C 5% CO_2_ incubator. The following morning cells were filtered and cultured at a concentration of 2E6 cells/ml at 37°C in 1 ml complete RPMI 1640 + 10% AB serum in a 24-well flat bottom plate. Cells were treated with different concentrations of anti-hCD40 antibodies or controls. After 48 h, 1 ml of complete RPMI 1640 with 10% AB serum and IL-2 (Proleukin, Sanofi) at a final concentration of 100 U/ml was added to each well. Half of the media was changed at day 4 and at day 6 adding fresh IL-2. At day 10, cells were harvested and washed twice in PBS with 2 mM EDTA. Cells were subsequently resuspended in complete RPMI 1640 + 10% AB serum in 50 ml tubes, counted and rested overnight at 37°C. The day after, cells were plated in a 96-well V-bottom plate in 200 µl volume per well and restimulated with 2 μM peptides or controls for 1 h at 37°C. After 1 h, Golgi Stop (BD, 51-2092KZ) and Brefeldin A (BFA) (BD, 420601) were added, and the cells were incubated for additional 4 h. Subsequently, cells were washed, and surface and intracellular staining was performed as described below in the *Intracellular Staining* methods section, and gating was on singlets, live cells, CD3^+^ followed by identification of TNFα^+^ and IFNγ^+^ in both CD4^+^/CD8^−^ and CD4^−^/CD8^+^ cells.

### Melanoma gp100 Peptide Presentation Studies

To detect processing and presentation of the melanoma gp100 Class I peptide gp100 209-217 in the context of cell surface HLA-A2, we used the TCR-like 1A9 mAb (15) H and L chain sequences from patent US20030223994A1 fused to a human IgG4 H chain with a C-terminal Dockerin V1 domain (6) and human L κ chain framework conjugated to either a Cohesin domain fused to a C-terminal CLIP tag (Sequence ID: AQS79240.1 residues 1–182 with appended GEPA) labeled with CLIP-647 according to the manufacturer’s protocol (NEB S9234), or a Cohesin-eGFP fusion protein (LDITH6 residues fused to a Cohesin domain from cellulosomal-scaffolding [Hungateiclostridium thermocellum] WP_065674352.1 residues 1044-1213 with a f1 flexible linker AVY25163.1 residues 580–608 to ABF13214.1 eGFP residues 123–361 followed by a EPEA sequence used for C-tag affinity matrix CaptureSelect™ [Thermo Fisher, 191307005]), or a Alexa Fluor^®^ 546 C_5_ Maleimide (ThermoFisher A10258) fused to Cohesin-SH (Sequence ID: ALO70206.1 residues 1–195 with a C61A replacement and CG appended) through maleimide reaction. The specificity of this antibody conjugate was confirmed by loading gp100 peptides (gp100 G9 Short [NH2]IMDQVPFSV[COOH] or gp100 G9 Long [NH2]CSSSKRIMDQVPFSV[COOH]) at 100 μM onto the human CD40^+^ HLA class-I A2^+^ lymphoblastoid JY cell line (ECACC^®^ 94022533-1VL) for 24 h and comparing, *via* flow cytometry, binding to loaded cells *versus* non-loaded cells (**Supplemental Figure 1A**). To deliver the peptide *via* DC-targeting, anti-hCD40-IgG4 mAbs were fused *via* the H-chain C-terminus to two copies of the short peptide interspersed between flexible linker sequences (7). A non-DC reactive hIgG4 mAb fused to the same gp100 peptide cassette was used as a non-targeting control. The gating strategy for flow cytometry analyses is described in **Supplemental Figure 1B**.

### ELISA

Cohesin fusion proteins were expressed in CHO-S cells from expression vectors containing the HIV-1 Env gp140 sequence (16) or Env fragments (gp120 residues 94–1550; gp41 residues 1551–2064, inserted between the Cohesin domain and 6 C-terminal His codons). ELISA plates were coated overnight at 4°C with 2 μg/ml of the Cohesin fusion proteins in 0.2 M sodium carbonate-bicarbonate buffer, pH 9.4. Serial dilutions of serum starting at 1:500 in TBS blocking solution (StartingBlock T20, Pierce or Thermo Fisher Scientific) were incubated in the wells overnight at 4°C. After washing, plates were incubated with HRP-conjugated goat anti-mouse IgG (Jackson ImmunoResearch, West Grove, PA, USA) in TBS blocking solution (Thermo Scientific, Rockford, IL, USA) for 2 h at 37°C, then washed and developed with HRP substrate (TMB, Life Technologies), stopped with equal volume of 1N HCl and read at 450 nm. The log10 transformed and normalized areas under the curves (AUC) data were calculated for each animal at each time point using GraphPad Prism 8 software. EC50 calculations were based on log10 transformed and normalized data with non-linear regression curve fit using sigmoidal dose response with variable slope constraints.

### Adoptive T Cell Transfer

CD4^+^ T cells specific to the Eα peptide bound to class II I-A^b^ were adoptively transferred, as previously described (17). Briefly, six skin-draining lymph nodes (LNs), spleens, and mesenteric LNs of TEα TCR transgenic mice were disrupted through a 40 µm cell strainer, washed with sterile Hanks’ Balanced Salt Solution (HBSS), and labeled with cell trace violet (CTV; Thermo Fisher Scientific), according to the manufacturer’s instructions. The cells were resuspended in sterile PBS at a concentration of 1E6 cells/ml, and 300 µl (3E5 cells) were injected intravenously into human CD40 mice (Taconic) (13). Twenty-four hours later, mice were injected intraperitoneally with 1 µg of anti-hCD40 11B6 non-covalently linked to Eα protein or 1 µg of anti-hCD40 11B6-CD40L non-covalently linked to Eα protein, and skin-draining LNs were harvested 4 days later. Cell suspensions were stimulated in complete RPMI 1640 with PMA (Sigma) (5 ng/ml) and Ionomycin (Sigma) (500 ng/ml) for 5 h. BFA (Biolegend) was added to the cell suspension 1 h after the beginning of the incubation. After stimulation, cells were washed and stained for surface markers as described below in the *Surface Staining for Flow Cytometry Analysis* methods section. Intracellular cytokine staining was performed with the BD Bioscience Cytofix/Cytoperm kit (BD Biosciences, San Jose, CA, USA), according to the manufacturer’s instructions and as described below. The Eα antigen was contained within 6xHis-Cohesin-TEα-Flex-v1C3 protein, which was expressed in *E. coli* as 6xHis-Cohesin (Sequence ID: ALO70206.1) fused at the C-terminus to residues 52–68 (ASFEAQGALANIAVD) of the I-Eα chain (Eα peptide), followed by KAGGASCQTPTNTISCTPTNNSNPKPCPAS.

### Cyclin D1 Tumor Curative Test

One million TNBC EO771.LMB Cyclin D1-expressing tumor cells (18) suspended in 20 μl sterile PBS were injected into the mammary fat pad of 10 to 15 weeks old female, human CD40 transgenic mice (Taconic). The mice were monitored daily by measuring tumor size with a caliper. At days 7, 17, and 24, mice were injected with 20 μg of anti-hCD40 (clone 11B6), anti-hCD40-CD40L (11B6-CD40L), IgG4 or IgG4-CD40L non-covalently linked to either Cyclin D1 constructs p1 or p2-4. The 20 μg dose was administered *via* injections of 10 μg vaccine, one to deliver p1 and the other to deliver p2-4. A group of mice was left unimmunized. On day 28, the mice were euthanized, and the tumors’ weight determined using an analytical scale. One Cohesin-human Cyclin D1 fusion protein was 6xHis-CthermoCohesin-Flex-v1-hCyclin D1-Peptide-1-f4-Ctag, which has a N-terminal Cyclin D1 segment (sequence ID: AAA36481.1 residues 1-48) appended onto the C-terminal Nhe I site of the His-tagged Cohesin coding sequence (6) followed by a flexible linker f4 (sequence ID: AJD85777.1 residues 697-793) terminating in EPEA, and was expressed in CHO-S cells and purified by C-tag affinity as described above. A second Cohesin-human Cyclin D1 fusion protein was 6xHis-CthermoCohesin-Flex-v1-hCyclin D1-Peptide-2-Peptide-3-Peptide-4-f4-Ctag, which has a Flex V1 sequence (Sequence ID: ALO70201.1 residues 466–496) appended onto the C-terminal Nhe/I site of the His-tagged Cohesin coding sequence (6) followed by the additional Cycln D1 segment (Sequence ID: NP_444284.1 residues 49–295) followed by a flexible linker f4 (Sequence ID: AJD85777.1 residues 697–793) terminating in EPEA, and was expressed and purified as described above.

### Surface Staining for Flow Cytometry Analysis

Cells were transferred to a 96-well V-bottom plate, washed twice in PBS, and incubated for 20 min at 4°C with Live/Dead™ Fixable Aqua Dead Cell Stain Kit (Thermo Fisher Scientific, Cat. L34965) or with Live/Dead-ef780 (Thermo Fisher Scientific 65-0865-14) following the manufacturer’s instructions. Cells were washed twice with PBS and incubated for 30 min on ice with the mix of antibodies in a volume of 50 μl. Finally, cells were washed and resuspended in 200 μl BD™ stabilizing fixative (BD Biosciences, Cat. 338036) per manufacturer’s instructions. Alternatively, cells were resuspended in a volume of 100 μl of Staining Buffer (PBS + 3% Bovine Serum + 5mM EDTA) in a U-bottom plate in the presence of Live/Dead-ef780 and the mix of antibodies for 30 min on ice. Cells were subsequently washed and resuspended in Staining Buffer before being analyzed. All analysis plots were pre-gated on singlet events and live. Cells were analyzed with a FACSCanto II or an LSRFortessa (BD Biosciences). Data was analyzed with FlowJo^®^ Software (TreeStar; Ashland, OR, USA).

The following antibodies were used for analysis of human DC activation: hCD80-PE, clone L307.4, ref 340294 (BD), hCD83-APC, clone HB15e, ref 551073 (BD); hCD86-FITC, clone 2331 (FUN-1), ref 555657 (BD); hCD11c-PE-Cyanine7, clone 3.9, ref 25011642, (eBioscience); hHLA-DR-V450, clone G46-6, ref 561359 (BD); hCD40-PE-Cyanine5, clone 5C3, ref 555590 (BD). The following panel was used in the melanoma gp100 peptide presentation assays: hCD83-BV421, clone HB15e, ref 562630 (BD); hCD86-Pe-Cy5, clone IT2.2, ref 555666 (BD); hCD11c-APC-Cyanine7, clone Bu15, ref 337218, (Biolegend); hHLA-DR-BV711, clone G46-6, ref 563696 (BD); hCD40-PE, clone 5C3, ref 555589 (BD) (used when the panel allowed it), hCD3-PE, clone SP34-2, ref 552127 (BD) or hCD3-FITC, clone HIT3a (RUO), ref 555339 (BD), hCD19-PE, clone HIB-19, ref 561741 (BD), or hCD19-FITC, clone 4G7, ref 347543 (BD), hCD14-PE, clone 61D3, ref 12-0149-42, (Thermo Fisher), or hCD14-FITC, clone M5E2, ref 301804 (Biolegend), hCD56-PE, clone B159, ref 555516 (BD) or hCD56-FITC, clone HCD56, ref 318304 (Biolegend), hCD66b-PE, clone G10F5, ref 561650 (BD) or hCD66b-FITC, clone G10F5, ref 305104 (Biolegend). hCD19-APC, clone HIB19, ref 555415 (BD) and hCD3-PerCP, clone SK7, ref 347344 (BD) were used in the human B cell proliferation panel. The following reagents were used for the adoptive T cell transfer assay: CD90.1-PerCPCy5.5 (BioLegend 202516), IFNg-PECy7 (Tonbo Biosciences 60-7311-U100), and CD4-BV711 (BioLegend 100447). The T cell proliferation panel was as follows: hCD3-BV711, clone UCHT1, ref 563725 (BD) or hCD3-PerCP clone SK7, ref 347344 (BD), hCD4-Pe-Cy7 clone SK3, ref 348789 (BD), hCD8-PacBlue clone 3B5, ref MHCD0828 (Invitrogen), hTNFα-APC clone RUO, ref 340534 (BD), and hIFNγ-PE clone RUO, ref 340452 (BD).

### Intracellular Staining

Human cells were stained for surface markers as described above. After 30 min incubation on ice with the antibodies for surface staining, cells were washed in PBS twice and resuspended in BD Cytofix/Cytoperm (BD Biosciences, 554714) for 20 min at 4°C, followed by three washes in 1X BD Permwash (BD Biosciences, 554714). Cells were subsequently incubated at room temperature covered by light in 1X BD Permwash with the antibody mix for intracellular cytokines. Following the incubation time, cells were washed three times in 1X BD Permwash and resuspended in BD™ stabilizing fixative diluted 1:3. Mouse cells were stained for surface markers as described above. After the incubation time, cells were washed with Staining Buffer and resuspended overnight with the mix of antibodies. Subsequently, cells were washed in BD Permwash, resuspended in BD Permwash, and analyzed. All analysis plots were pre-gated on singlet events and live. Cells were analyzed with a FACS Canto II or an LSR Fortessa (BD Biosciences). Data were analyzed with FlowJo^®^ Software.

### Mouse Experiments

Human CD40 transgenic mice have the human CD40 region BAC inserted into a wild type C57BL/6 background [Taconic strain 12692 (13)]. CD90.1 congenic TEα Rag1^−/−^ CD4 TCR transgenic mice to I-Eα_52−68_ on the C57BL/6 background were obtained from M. Jenkins (University of Minnesota). All experiments were performed with 6- to 12-week-old female and male mice. Mice were housed in micro-isolator cages and fed irradiated food and acidified water. The Baylor Scott and White Research Institute and South Dallas Veteran’s Administration Institutional Care and Use Committees approved all mouse protocols.

### Statistical Analyses

Data are presented as means ( ± SEM). Statistical significance was determined by Student’s t test with or without Welch’s correction. A P value < 0.05 was considered as statistically significant. GraphPad Prism^®^ software was used for statistical calculations. The following nomenclature was used in figures to indicate the statistical significance: * P 0.01 to 0.05; ** P 0.001 to 0.01; *** P 0.0001 to 0.001; **** is P< 0.0001.

## Results

### Fusing Various Antigens to Agonistic Anti-CD40 mAbs Can Dull CD40 Activation Efficacy, but Some Anti-CD40 mAb-Antigen Fusions Synergize With sCD40L to Restore CD40 Activation Potency

**Figure 1:**
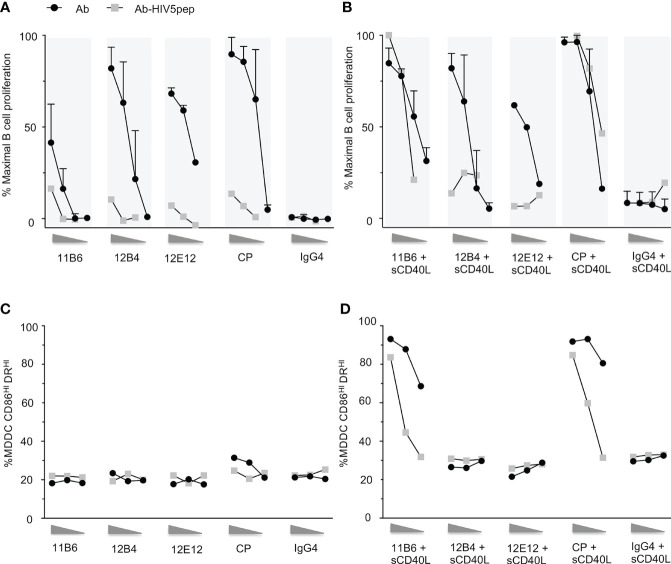
Response of human B cells and human MDDCs to a dose range of anti-CD40 mAbs and matched anti-CD40 HIV5pep antigen fusion proteins incubated with and without a constant low dose of soluble CD40L. **(A)** shows responses to dose ranges (shown left to right: 10, 1, 0.1 nM for the three point titrations, and 10, 1, 0.1, 0.01 nM for the four point titrations) in six collated independent experiments to the indicated anti-CD40 or control hIgG4 antibodies or of one experiment with the same anti-CD40 hIgG4 or control hIgG4 antibody fused to the HIV5pep antigen. **(B)** shows response of four collated independent experiments to doses (shown left to right: 10, 1, 0.1 nM for the three point titrations, and 10, 1, 0.1, 0.01 nM for the four point titrations) of the same anti-CD40 or control antibodies and one independent experiment with matching HIV5pep fusion proteins in the presence of 100 ng/ml (6 nM) monomeric soluble human CD40L. The data were normalized to the maximum B cell proliferation observed in each experiment. **(C, D)** show DC activation responses to a dose range (shown left to right: 10, 1, 0.1 nM) of each indicated anti-CD40 hIgG4 mAb and matching anti-CD40-HIV5pep fusion protein antibodies in the absence **(C)** or presence of 100 ng/ml (6 nM) soluble human CD40L **(D)**. The percentage of cells with high expression of CD86 and DR determined by flow cytometry is shown. Data are from a single representative experiment. We have previously shown analogous data for the indicated non-fusion antibodies with or without sCD40L (13), and these data are included for reference. The control hIgG4 antibody we used does not bind to human monocytes, B cells, or MDDCs (7).

Fusion of antigens to the C-terminus of chimeric or humanized anti-CD40 agonistic antibodies can dull or eliminate the agonistic property of the parent antibody (7). We generated a panel of agonistic anti-CD40 antibodies configured on human IgG4 isotypes, with or without a concatenated string of HIV-1 long T cell epitope-rich peptides from the Gag, Nef, and Pol gene regions grafted to their H chain C-termini (7). It is known that antibody isotype can impact CD40 agonistic potency *via* FcRγ interaction (2); thus, the human IgG4 isotype was selected since it has low binding to FcRγ (19), allowing a clear interpretation that the agonistic and antigen-presenting properties of our tested panel of anti-CD40-antigen fusion mAbs was due exclusively to CD40 interaction. As we described previously (13), the four selected parental mAbs, all with their variable regions configured as human IgG4 H chain and human K L chain constant regions, varied in their potency for evoking human B cell proliferation over a 100-fold range of agonist efficacies (**Figure 1A**) with rank order CP>12B4≥12E12>11B6 [CP is Pfizer CP-870,893, an agonist anti-CD40 human IgG2 antibody in clinical development, here re-configured as a human IgG4 antibody (20)]. In all cases, the agonist activity was severely decreased (>10–100-fold) when the mAb C-terminus was fused to five concatenated HIV-1 long peptide regions interspersed with glycosylated flexible linkers (HIV5pep antigen cassette) (**Figure 1A**). We also previously showed that the anti-CD40 11B6 and CP mAbs, which do not significantly block CD40L interaction with CD40, synergize strongly with soluble CD40L (13) (**Figure 1B**). The agonistic anti-CD40 11B6, 12B4, 12E12, and CP hIgG4 mAbs all became very weak agonists for eliciting B cell proliferation when the HIV5pep antigen cassette was grafted to their H chain C-termini (**Figure 1A**). Addition of suboptimal levels of monomeric sCD40L restored the B cell proliferation potency of the anti-CD40 11B6-HIV5pep and anti-CD40 CP-HIV5pep mAbs to the level characteristic of the analogous “naked” anti-CD40 11B6 and CP mAbs incubated with sCD40L, but had no significant effect on the anti-CD40 12B4-HIV5pep or anti-CD40 12E12-HIV5pep mAbs (**Figure 1B**).

Since dendritic cells (DCs) are the desired target cells for delivering antigens for potent vaccination responses, we examined these same four agonist anti-CD40-HIV5pep fusion proteins for potency of upregulation of activation markers on human myeloid-derived dendritic cells (MDDCs). As previously described (13) and as observed with the B cell proliferation assay (**Figures 1A, B**), the non-fusion antibody panel had minimal efficacy at the 10–0.1 nM concentrations tested compared to the more sensitive B cell proliferative response (**Figure 1C**). However, as previously observed (13), when assayed over this dose range together with a suboptimal dose of soluble monomeric CD40L (sCD40L), there was no effect on the dose response of the anti-CD40 12B4 and anti-CD40 12E12 antibodies, but the low 6 nM dose of sCD40L greatly increased the potency and efficacy of the anti-CD40 11B6 and CP antibodies (**Figure 1D**). As observed with B cell proliferation, suboptimal level of sCD40L also potentiated the activity on MDDCs of the anti-CD40 11B6-HIV5pep and anti-CD40 CP-HIV5pep fusion proteins, but had no effect at the tested doses on the anti-CD40 12B4-HIV5pep or anti-CD40 12E12-HIV5pep fusion proteins (**Figure 1D**). Overall, the extent of sCD40L potentiation at the sCD40L concentration tested was ~5- to 10-fold less with the anti-CD40-HIV5pep fusion proteins than that observed with the anti-CD40 11B6 and CP non-antigen fused mAbs (**Figure 1D**).

Unlike in the B cell proliferation assay, a large increase in efficacy (i.e., maximal activation marker increase achieved) between antibody alone and antibody with sCD40L was observed, indicating a greater potential for co-operation between these two CD40 agonist types on MDDCs. This effect was also seen with potentiation of cytokine secretion by non-antigen fused anti-CD40 11B6 and CP antibodies when combined with sCD40L (13).

### Agonistic Properties of Anti-CD40 mAbs Dulled by Antigen Fusion Can Be Restored *via* Direct Fusion of CD40L to Their Light Chain C-Termini

**Figure 2:**
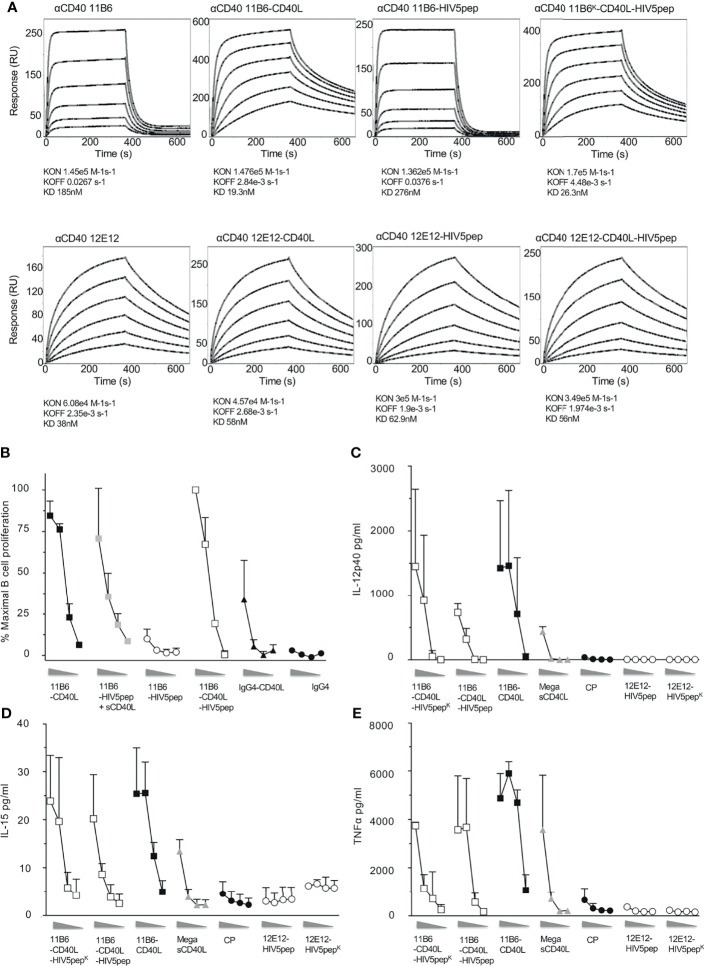
Direct fusion of CD40L to anti-CD40 antibody-antigen fusion protein improves affinity for CD40 binding and confers high agonist activity on human B cells and MDDCs. **(A)** Shows surface plasmon resonance analyses for anti-CD40 11B6 (top panels) and anti-CD40 12E12 (bottom panels), with or without directly fused CD40L and/or with or without the HIV5pep adduct. **(B)** Shows B cell proliferation in response to a dose range (left to right, 10, 1, 0.1, 0.01 nM) of the indicated anti-CD40 11B6 hIgG4 antibodies or antibody-HIV5pep fusions with added 60 nM sCD40L, or with CD40L directly fused to the antibody L chain C-termini. Data represent averaged values for two donors each normalized for maximum proliferation. **(C–E)** Shows DC activation to a dose range (left to right, 10, 1, 0.1, 0.01 nM) of each indicated IgG4 mAb or Mega sCD40L (a trimeric form of sCD40L, EnzoLifeScience, ref ALX-522-110-C010) based on the extent of cytokine secretion determined at 24 h. The results presented are averaged data over three experiments using two different donors. Panels **(C–E)** show, respectively, the averaged IL-12 p40, TNFα, and IL-15 secretion. The error bars are standard deviation of the mean. **Supplemental Table 1** summarizes the relative efficacies of this cytokine secretion data.

We have shown that direct fusion of CD40L to agonistic anti-CD40 mAbs stabilizes binding to CD40 and thus potentiates their agonist activities, emulating or surpassing the synergism seen with co-administered sCD40L (13). While previous prototypes of anti-CD40-HIV5pep vaccines were based on the humanized 12E12 mAb (14, 21), we focused on the anti-CD40 11B6 mAb since it shows the strongest cooperation with soluble CD40L for agonist activity (**Figures 1B, D**) (13). Similarly to what we previously observed (13), using Surface Plasmon Resonance analyses with solid phase CD40 ectodomain to test the binding kinetic properties of our anti-CD40 constructs, direct fusion of CD40L to the anti-CD40 12E12 did not improve the affinity of this antibody, likely due to the fact that the anti-CD40 12E12 binding site interferes with CD40L binding, but in contrast, CD40L fusion greatly improves the affinity *via* stabilizing off-rate of the anti-CD40 11B6 antibody, which binds CD40 without blocking CD40L access (**Figure 2A**) (13). The direct fusion of the HIV5pep adduct on both the anti-CD40 12E12 and on the anti-CD40 11B6 slightly reduced the affinity of both antibodies by ~66% and ~50% respectively, likely due to changes in diffusion from increased bulk. However, direct fusion of CD40L to the anti-CD40 11B6-HIV5pep construct maintained the greatly increased affinity to CD40 observed with anti-CD40 11B6-CD40L without fused antigen, while anti-CD40 12E12-HIV5pep did not improve affinity to CD40 after direct fusion to CD40L (**Figure 2A**). These data confirm that the direct fusion of CD40L on anti-CD40 11B6, either with or without an antigen attached, improves the affinity of the antibody for CD40.

Thus, we asked if the improved binding to CD40 by anti-CD40 11B6-CD40L HIV5pep fusion protein could mirror the restoration of high agonist activity we observed by adding soluble CD40L. In B cell proliferation assays, anti-CD40 11B6-HIV5pep had minimal agonist activity; however, both anti-CD40 11B6-HIV5pep + sCD40L and anti-CD40 11B6-CD40L-HIV5pep had high agonist activity that approached that of the superagonist anti-CD40 11B6-CD40L (**Figure 2B**). This indicates that direct fusion of CD40L can render a weak agonist anti-CD40-HIV5pep fusion construct into a strong agonist. Anti-CD40 11B6-CD40L-HIV5pep constructs also had potent agonist activity on DCs (**Figures 2C–E**), exceeding the activity of the naked anti-CD40 CP agonist hIgG4 mAb or the highly active Mega sCD40 trimer (22). Thus, the observed increase in CD40 binding by the anti-CD40 11B6-CD40L-HIV5pep protein is associated with highly increased activity on B cells and DCs.

**Figure 3:**
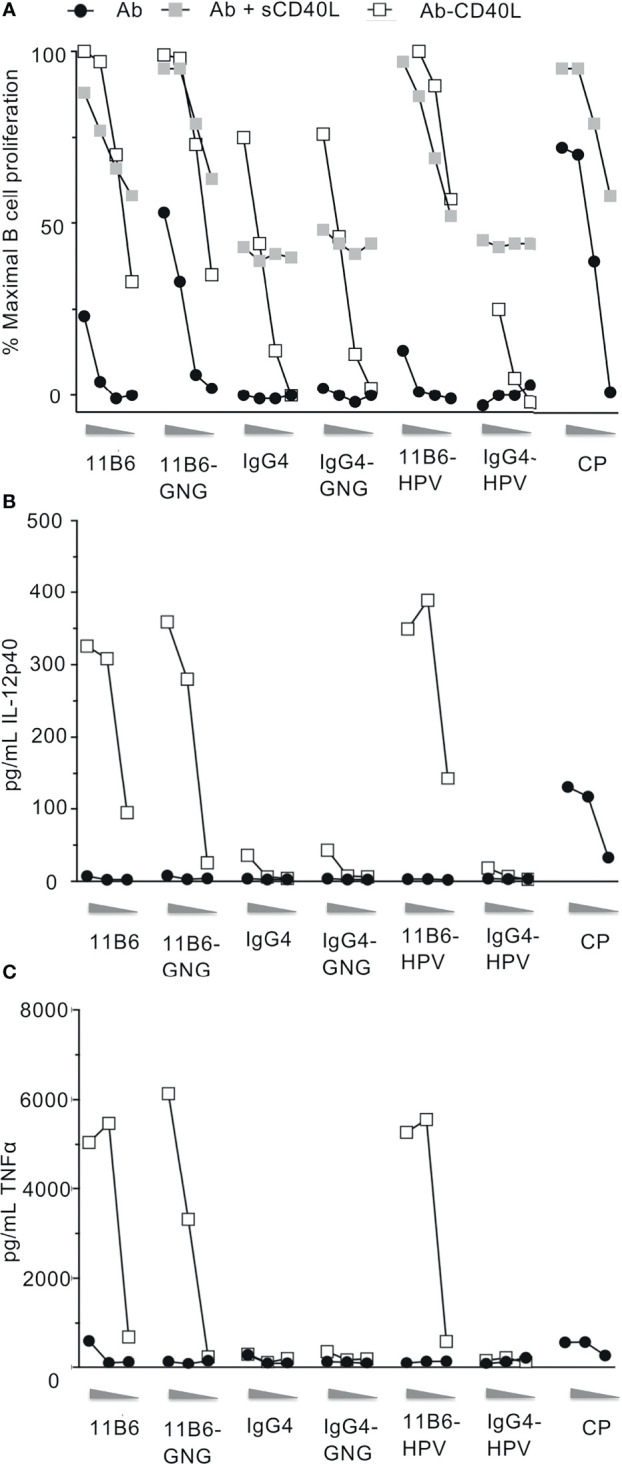
Anti-CD40 11B6-CD40L fused to HIV-1 Gag p24, Nef, and Gag p17 or HPV16 E6/E7 antigens is highly active on human B cells and DCs. **(A)** Shows B cell proliferation in response to a dose range (shown left to right: 10, 1, 0.1, 0.01 nM) of the indicated antibody or antibody-antigen fusion protein; curves with gray filled square symbols are responses doses to the indicated antibody or anti-CD40 11B6-antigen fusion protein in the presence of 1 μg/ml (60 nM) soluble human CD40L or fused directly to CD40L. Data represent a single experiment normalized for maximum proliferation (95%) *versus* baseline proliferation without antibody (10%). **(B, C)** show MDDC cytokine secretion responses to a dose range (shown left to right: 10, 1, 0.1 nM) of each indicated mAb. Data show observed concentration values in pg/ml. The maximal values were IL-12p40, 359 ng/ml; TNFα, 6134 ng/ml. A dose range of the agonistic CP hIgG4 antibody is included for reference.

To test if CD40L fusion to anti-CD40 IgG4 L chain C-termini could also increase agonist potency while fused to other desirable antigens, we compared agonistic activities when anti-CD40 11B6 and 12E12 IgG4 mAbs were fused at their H chain C-termini to concatenated HIV-1 Gag p24 Nef Gag p17 (called GNG) or HPV16 E6/E7 (called HPV) antigens (9), with or without CD40L directly fused to the L chain. These two antigens do not significantly dull the already low potency of B cell CD40 activation of the parent 11B6 mAb, but CD40L L chain fusion potentiated the activation to levels equal to the mAbs co-administered sCD40 (**Figure 3A**). The increase on B cell proliferation activity was mirrored by robust cytokine production from MDDC elicited by anti-CD40 11B6-GNG (**Figures 3B, C**). Altogether, these data demonstrate the superagonist properties of anti-CD40 11B6-CD40L on B cells and DCs are maintained when fused to the three tested antigen modules—HIV5pep, GNG, and HPV16 E6/E7.

### Anti-CD40-CD40L-Antigen Fusion Vaccines Preferentially Expand Antigen-Specific Memory CD8^+^ T Cells *In Vitro*

**Figure 4:**
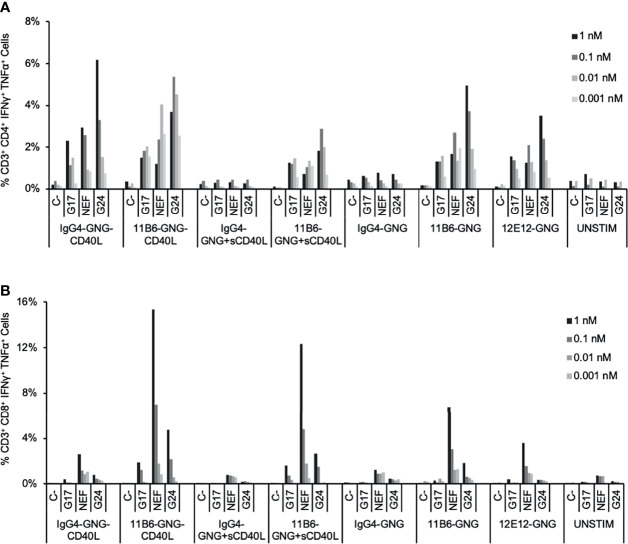
Expansion by CD40-targeted GNG antigen of HIV-1-specific T cells in HIV-1-infected donor PBMC cultures. HIV-1^+^ donor PBMCs were cultured with a dose range of anti-CD40-GNG fusion proteins with and without a low dose of sCD40L (100 ng/ml; 6 nM) and IL-2 for 9 days, followed by stimulation with peptides for pools HIV-1 Gag p17, Nef, and Gag p24 for 6 h, then analyzed by ICS. The data show the percentage at the end of the culture of antigen-specific **(A)** CD4^+^ and **(B)** CD8^+^ T cells producing IFNγ + TNFα in response to peptide stimulation. Similar data were obtained in a replicated experiment (**Supplemental Figure 2**).

Peripheral Blood Mononuclear Cell (PBMC) and DC-T cell co-culture systems are useful in *in vitro* assays for validating DC-targeting prototype vaccine constructs, in particular for selecting the best DC receptor to target, e.g., for cellular T cell response (8), as well as confirming the efficacy of the selected fused antigen for eliciting a broad range of T cell peptide specificities for both CD4^+^ and CD8^+^ T cell recall responses across a range of HLA types (7). To investigate the potential impact of associating antigen delivery with potent CD40 activation, we first evaluated the efficacy for HIV-1 antigen-specific T cell expansion in HIV-1^+^ donor PBMC cultures of anti-CD40 mAbs fused at the C-termini to a Gag p17-Nef-Gag p24 (GNG) antigen cassette, with and without a low dose of sCD40L or with CD40L directly fused to the L chain C-terminus. PBMCs from the donor shown in **Figure 4** treated with a dose ≥0.01 nM of CD40-targeting GNG constructs expanded memory CD4^+^ T cells specific to epitopes within all three Gag p17, Nef, and Gag p24 regions, while such cells were minimally represented in antigen non-stimulated or non-targeting hIgG4-GNG treated cultures (**Figure 4A**). All anti-CD40-GNG targeting constructs gave similar CD4^+^ T cell GNG-specific responses, except anti-CD40 11B6-CD40L-GNG elicited greater GNG-specific responses at the lowest tested (0.001 nM or 1 pM) dose consistently in two donors tested, although these were not statistically significant (**Figure 4A** and **Supplemental Figure 2A**). In this donor, all anti-CD40-GNG targeting constructs elicited CD8^+^ T cell responses specific to epitopes, especially within Nef, while such cells were minimally represented in antigen non-stimulated or non-targeting hIgG4-GNG treated cultures (**Figure 4B**). The most robust expansion of Nef-specific CD8^+^ T cells was observed in cultures treated with 1 and 0.1 nM of the highly activating anti-CD40 11B6-CD40L-GNG and anti-CD40 11B6-GNG + sCD40L constructs (**Figure 4B** and **Supplemental Figure 2B**).

The improved antigen-specific CD8^+^ T cell response associated with concomitant HIV-1 antigen delivery and activation *via* CD40 we observed above with the GNG model antigen suggested the possibility of a similar benefit for CD40 targeting of the concatenated HIV5pep antigen cassette that is well advanced towards clinical development (7, 21). HIV5pep was designed to incorporate relatively conserved CD4^+^ and CD8^+^ T cell epitopes across multiple HLA haplotypes from the HIV-1 Gag, Nef, and Pol regions within five peptides. We compared *via in vitro* PBMC expansion cultures various formats of anti-CD40 11B6-CD40L-HIV5pep prototype vaccines compared to the anti-CD40 12E12-HIV5pep Gen 2 vaccine (14). In the Gen 1 format, the five HIV-1 peptide regions intercalated with glycosylated flexible linkers are grafted to the mAb H chain C-termini, and this is compatible with adding CD40L to the available L chain C-termini. In the Gen 2 format, the same long peptides and linkers are used, but two are now grafted to the L chain C-termini. By configuring the same 2 + 3 peptides and linkers on separate mAb H chains using knob-in-hole (KIH) technology (23), highly activating anti-CD40 11B6-CD40L-HIV5pep KIH was also available (**Figure 2B**). Previous studies demonstrated that the Gen 1 and Gen 2 formats yield identical *in vitro* HIV-1 antigen-specific T cell responses (14). The Gen 1 and Gen 2 versions of the HIV5pep vaccine product were unsuitable for Good Manufacturing Practice (GMP) scale-up due to fragmentation and aggregation issues that were not evident at the research scale; the KIH Gen 3 product was developed to circumvent these problems and is now in GMP production.

**Figure 5:**
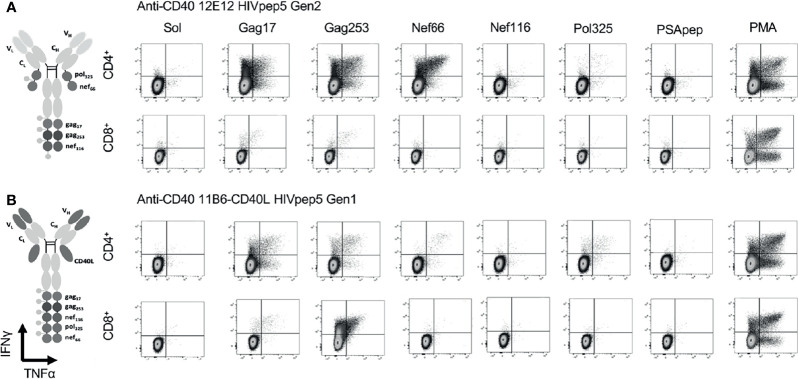
CD40-targeted HIV5pep antigens with and without fused CD40L tested *via in vitro* expansion of HIV-1-specific T cells in HIV-1-infected donor PBMC cultures. HIV-1^+^ donor 1 PBMCs were cultured for 9 days with IL-2 and anti-CD40 HIV5pep fusion proteins (1 nM), followed by stimulation with long peptides specific for each of the five HIV-1 Gag, Nef, and Pol regions for 6 h with Golgi Stop + BFA, then analyzed by ICS. This is IHC data from one experiment with two of the four indicated proteins tested on one of the donors shown in **Figure 6**. PSApep and sol indicate, respectively, non-relevant peptide and solvent only negative controls, and PMA represents polyclonal stimulation by Phorbol 12-Myristate 13-Acetate and Ionomycin. Minimal responses to specific peptides were observed in cultures expanded without vaccine stimulation (not shown) or with hIgG4-HIV5pep non-targeting control protein (7). **(A)** shows responses elicited by anti-CD40 12E12 HIV5pep Gen 2, and **(B)** shows responses elicited by anti-CD40 11B6-CD40L-HIV5pep Gen 1. Previous data show that Gen 1 *versus* Gen 2 HIV5pep configuration on anti-CD40 12E12 elicit identical ranges and extent of responses in similar experiments (14). **Supplemental Figure 3** shows similar data for anti-CD40 12E12-HIV5pep Gen3 *versus* anti-CD40 11B6-CD40L-HIV5pep Gen3 responses in Donor 3 as a more direct comparison with matching Gen3 formats.

**Figure 6:**
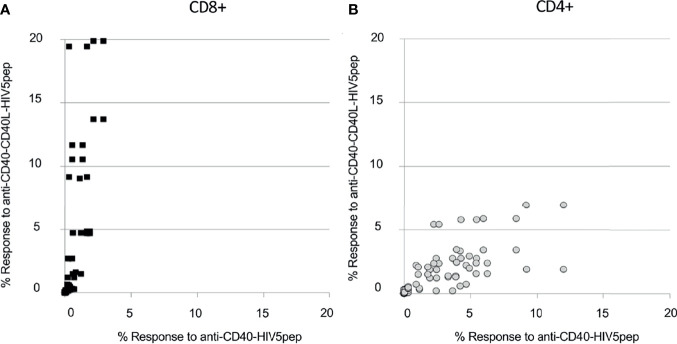
Anti-CD40-CD40L targeted HIV5pep antigens preferentially expand many HIV-1-specific CD8^+^ T cell responses in HIV-1-infected donor PBMC cultures. HIV-1^+^ donor PBMCs were cultured for 9 days with IL-2 and anti-CD40 HIV5pep fusion proteins (1 nM; 2 nM for KIH proteins), followed by stimulation with long peptides specific for each of the five HIV-1 Gag, Nef, and Pol regions for 6 h with BFA, then analyzed by ICS. This is collated data from experiments with each of the indicated protein pairs tested on four donors. Each point represents the % value for IFNγ^+^ + TNFα^+^ HIV5pep-specific CD8^+^ [panel **(A)**, black filled squares] or CD4^+^ [**(B)**, gray circles] T cells for each response specific to each long peptide elicited in each donor comparing anti-CD40 11B6-CD40L-HIV5pep KIH Gen 2 and anti-CD40 11B6-CD40L-HIV5pep Gen 1 (Y axis) to anti-CD40 12E12-HIV5pep KIH Gen 2 and anti-CD40 12E12-HIV5pep Gen 2 (X axis). The fold difference between the antigen-specific CD8^+^ T cell responses elicited by anti-CD40 11B6-CD40L *versus* anti-CD40 12E12 was on average 7.2X, with a minimum of 0.3X and a maximum of 61X, calculated on 36 coupled values in which 30 had a greater response to anti-CD40 11B6-CD40L. For this calculation, the threshold was 0.5% response in at least one of the coupled values. **(B)** Shows the whole dataset. The fold difference between the antigen-specific CD4^+^ T cell responses elicited by anti-CD40 11B6-CD40L *versus* anti-CD40 12E12 was on average 0.7X, with a minimum of 0.15X and a maximum of 2.4X, calculated on 44 coupled values in which 34 had a greater response to anti-CD40 12E12. For this calculation, the threshold was 0.5% in the response in both of the values in the couples.

We compared *in vitro* T cell responses to anti-CD40 12E12-HIV5pep Gen 2 to the highly activating anti-CD40 11B6-CD40L-HIV5pep Gen 1, and both vaccines elicited CD4^+^ T cell responses specific to epitopes with the Gag17, Gag253, Nef66, and Pol325 long peptide regions, as well as CD8^+^ T cell responses within the Gag17 and Gag253 regions in the illustrated donor (**Figure 5**). However, the highly activating anti-CD40 11B6-CD40L-HIV5pep vaccine preferentially expanded the dominant Gag253-specific CD8^+^ T cell response, while the same CD4^+^ T cell responses to Gag17, Gag253, Nef66 were elicited, but to a lesser abundance (**Figure 5B**). Comparative *in vitro* T cell expansion studies with anti-CD40-HIV5pep *versus* anti-CD40-CD40L-HIV5pep constructs were replicated in three other HIV-1^+^ donors and are collated (**Figure 6**). Overall, T cell responses were highly correlated between the vaccine pairs (Pearson’s test 0.85 for CD8^+^ and 0.7 for CD4^+^) indicating the vaccines presented a similar array of epitopes for activating populations of antigen-specific T cells. However, the HIV-1-specific CD8^+^ T cell responses were substantially (7.2X) more robust with the highly activating anti-CD40-CD40L-HIV5pep vaccines (**Figure 6A**). In contrast, the HIV-1-specific CD4^+^ T cell responses were similar or slightly depressed (0.7X) with the highly activating anti-CD40-CD40L-HIV5pep vaccines compared to anti-CD40-HIV5pep (**Figure 6B**), likely reflecting the increased dominance of antigen-specific CD8^+^ T cells in these cultures.

**Figure 7:**
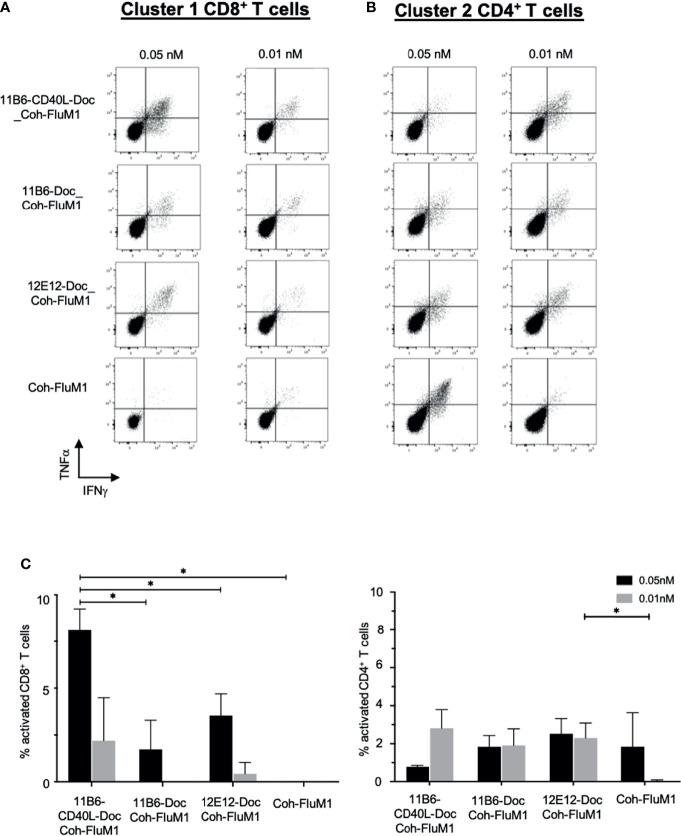
Anti-CD40-CD40L-targeted Flu M1 antigen preferentially expands Flu M1-specific CD8^+^ T cell responses at low doses in normal donor PBMC cultures. Normal donor PBMCs were cultured for 9 days with IL-2 and a dose range of anti-CD40 vehicles in complex with Flu M1 protein, followed by stimulation with Flu M1 peptide pools for 6 h with Golgi Stop and BFA, then analyzed for intracellular IFNγ^+^ and TNFα^+^ by ICS. Results for one representative experiment for CD8^+^ T cells stimulated with cluster 1 **(A)** and CD4^+^ T cells stimulated with cluster 2 **(B)** are shown. **(C)** shows repeated experiments with this donor treated with 0.05 and 0.01 nM of the different anti-CD40 vehicles in complex to Flu M1 protein or controls (n=4 for 0.01 nM and n=3 for 0.05 nM); data are represented as n – untreated cells. In all cases anti-hCD40 11B6-CD40L gave more robust CD8^+^ T cell responses at these low concentrations (mean = 9.1% ± 2 at 0.05 nM and = 3.8% ± 4.4 at 0.01 nM) than the other conditions (mean = 2.9% ± 2.4 at 0.05 nM and = 1.3% ± 0.6 at 0.01 nM). Previous studies [(6); **Figure 5** ] have demonstrated that additional Coh-Flu M1 and hIgG4-Doc: Coh-Flu M1 non-targeting controls gave similar minimal responses compared to CD40-Doc: Coh-Flu M1. **Supplemental Table 2** shows in detail each Influenza Matrix 1 peptide used and how they are grouped into three clusters. * indicates a statistically significant difference of P=0.01-0.05.

To extend our observations with HIV-1 donor PBMCs and to further validate the importance of our vaccine platform technology, we compared the ability of anti-CD40-CD40L to target Influenza matrix 1 (Flu M1) protein and expand Flu M1-specific memory T cells. PBMCs obtained by apheresis of Normal Donor (ND) (All Cells, CA. ID:11588) were cultured with dose ranges of Cohesin-Flu M1 alone (Flu M1) or in complex with three different CD40-targeting antibody vehicles. We have previously described a convenient method for non-covalent assembly of anti-DC antibodies and antigens using a bacterial Dockerin (Doc) domain fused to the antibody heavy chain C-terminus, and antigen such as Flu M1 fused to a Cohesin (Coh) counter-domain (6). After an expansion culture period of 10 days, cells were harvested and restimulated with three pools (clusters) of overlapping 15 mer peptides covering the entire Flu M1 protein, then analyzed by ICS for peptide-elicited production of intracellular IFNγ and TNFα. As represented by the donor shown in **Figure 7** and statistically verified by four replicated experiments, compared to Flu M1 alone, anti-CD40-Flu M1 complexes efficiently expanded CD8^+^ T cells specific to cluster 1, with anti-CD40 11B6-CD40L showing a significance advantage at the 50 pM dose (p=0.018) (**Figures 7A, C**). Anti-CD40 11B6-CD40L was more potent at the low concentrations tested (10 and 50 pM) compared to the other anti-CD40 antibodies, with a significant difference at 50 pM compared to anti-CD40 11B6 (p=0.035). In these same cultures, anti-CD40 11B6 and anti-CD40 12E12 vehicles were similarly potent at expanding Flu M1-specific CD4^+^ T cells to cluster 2 and 3 peptides, but these responses were less robust with anti-CD40 11B6-CD40L targeting (**Figures 7B, C**), likely due to the dominant expansion of Flu M1-specific CD8^+^ T cells in these cultures. All the three anti-CD40 targeting agents at the higher 50 pM dose gave reduced or equal Flu M1-specific CD4^+^ T cell expansion compared to Flu M1 alone (**Figures 7B, C**), but this effect was not observed at the lower 10 pM dose or with Flu M1-specific CD8^+^ T cell expansion (**Figures 7A, C**). Repeated experiments with this donor confirmed these results (**Figure 7C**). These data, together with what we observed with GNG and HIV5pep as antigens, demonstrate the high potency of the anti-CD40 11B6-CD40L-antigen at the lower doses tested and its improved efficacy to preferentially expand CD8^+^ T cells compared to anti-CD40 12E12-antigen.

### Anti-CD40 11B6-CD40L-Melanoma gp100 Antigen Targeted to MDDCs Presents the Immune-Dominant Class I Peptide 209-217 More Efficiently Than Anti-CD40-Melanoma Antigen Fusions Without Linked CD40L

**Figure 8:**
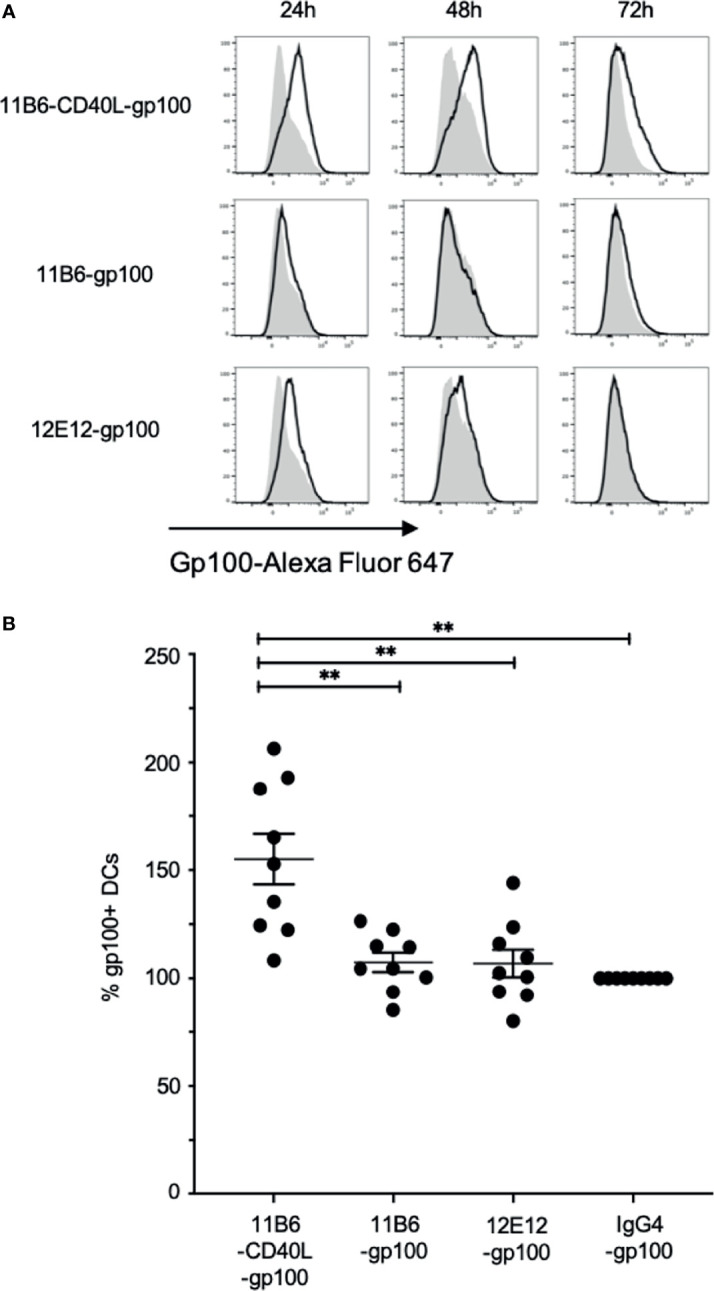
Anti-CD40-CD40L-targeted melanoma gp100 antigen is efficiently presented on MDDC HLA-A2. Normal donor MDDCs were treated with 50 nM anti-CD40 or control hIgG4 antibodies fused to a dominant Class I gp100 peptide. Cultures were harvested at 24, 48, and 72 h and probed with fluorescently tagged 1A9 mAb to detect surface gp100 peptide/HLA-A2 complex *via* flow cytometry analysis. **(A)** The panels show frequency *versus* fluorescence intensity plots of MDDCs treated with either the indicated anti-CD40-gp100 constructs (black outline), or control hIgG4-gp100 (gray areas). The data are one representative of three independent experiments. **(B)** Total fluorescence of each anti-CD40-gp100-treated population was calculated and compared to the hIgG4-gp100-treated cells, which was set at 100%. Averaged over the three experiments and over the three time points anti-CD40 11B6-CD40L-gp100 gave a mean value of 155 ± 35; anti-CD40 11B6-gp100 mean = 107 ± 14; anti-CD40 12E12-gp100 mean = 107 ± 19. The overall values for anti-CD40 11B6-CD40L-gp100 were significantly greater compared to 11B6-gp100 (p = 0.0032), 12E12-gp100 (p = 0.0034), and hIgG4-gp100 (p = 0.0015). ** indicates a statistically significant difference of P=0.001-0.01.

To further investigate the improved CD8^+^ responses, we used the 1A9 mAb (15) to detect processing and presentation on MDDCs of the melanoma gp100 Class I peptide gp100 209-217 in the context of cell surface HLA-A2. Indeed, the size of the CD8^+^ T cell responses is known to correlate with the duration of the antigen presentation (24). To deliver the peptide *via* DC-targeting, anti-human CD40 IgG4 mAbs with and without directly linked CD40L were fused *via* the H chain C-terminus to two copies of the peptide interspersed between flexible linker sequences (7). These constructs at 50 nM were incubated with MDDCs, and the cells were probed with the labeled 1A9 mAb after 24, 48, and 72 h and analyzed by flow cytometry. In three independent experiments, compared to anti-CD40 11B6-gp100 or anti-CD40 12E12-gp100-loaded MDDCs, anti-CD40 11B6-CD40L-gp100-loaded MDDCs had greater and more sustained specific cell surface reactivity to the 1A9 mAb, which correlates with the observed increase in expression of surface activation markers CD83 and CD86 (**Figure 8** and **Supplemental Figure 1C**). These results suggest that anti-CD40 11B6-CD40L-mediated antigen delivery to DCs can prolong antigen presentation on MHC I, potentially explaining the observed increased CD8^+^ T cell responses to anti-CD40 11B6-CD40L delivery.

### Anti-CD40 11B6-CD40L-Antigen Vaccine Platform Augment Adjuvant-Free *In Vivo* Immune Responses in Mouse Models

**Figure 9:**
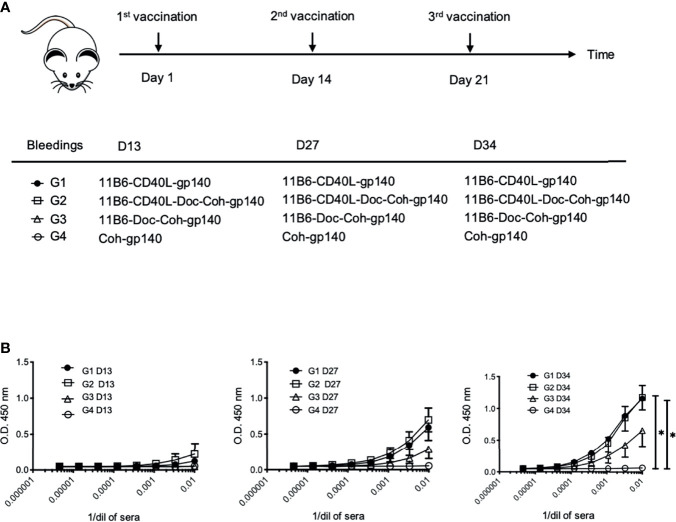
CD40-targeted HIV-1 gp140 antigen with fused CD40L elicits superior gp140-specific serum IgG responses in human CD40 transgenic mice. **(A)** Human CD40 homozygous transgenic mice were vaccinated *via* intraperitoneal injection with (Group 1) anti-CD40 11B6-CD40L directly fused to HIV-1 gp140, (Group 2) anti-CD40 11B6-CD40L-Dockerin + Cohesin-gp140, (Group 3) anti-CD40 11B6-Dockerin + Cohesin gp140, or (Group 4) Cohesin-gp140. Dose was normalized to be the molar equivalent of 1 μg of the Group 2 vaccine, except for the non-targeting control Group 3 (2 μg or ≅3 molar equivalents). Vaccination was at Day 1, Day 14, and Day 21 with small blood draws taken at Day 13 (D13), Day 27 (D27), and Day 34 (D34). **(B)** The graphs show serial dilutions of sera analyzed for anti-gp140 IgG reactivity by ELISA as described in (16) except that anti-mouse IgG-HRP reagent was the detecting reagent. Group sizes were G1, n=3 and G2-4, n=4. Directional error bars are S.E. of the mean. * indicates a statistically significant difference of P=0.01-0.05.

To test the potential of anti-CD40 11B6-CD40L-antigen constructs for increasing vaccine efficacy in the absence of co-administered adjuvant, human CD40 transgenic mice were vaccinated with anti-CD40 11B6 hIgG4 delivery vehicles, with and without fused CD40L, coupled to HIV-1 Env gp140. Vaccination with anti-CD40 11B6-CD40L directly fused to gp140 was compared to vaccination with anti-CD40 11B6-CD40L non-covalently coupled to gp140 *via* a Dockerin-Cohesin interaction (6), to anti-CD40 11B6 non-covalently coupled to gp140. Cohesin-gp140 was used as the non-targeted control (**Figure 9A**). Both anti-CD40 11B6-CD40L non-covalently coupled to gp140 and anti-CD40 11B6-CD40L directly fused to gp140 elicited serum anti-gp140 IgG titers that were detected as early as 13 days after a single vaccination (**Figure 9B**, left panel). Responses to these anti-CD40 11B6-CD40L-based vaccines increased to similar extents when monitored 13 days after each of two subsequent vaccinations administered at 2-week intervals (day 14 and day 21) after the initial D0 vaccination. These responses were significantly increased compared to the group immunized with the non-targeted gp140 after the third vaccination (p<0.05) (**Figure 9B**, right panel) where non-targeted control Cohesin-gp140 failed to elicit any detectable anti-gp140 IgG responses even after three vaccinations despite using a three-fold molar excess of gp140 compared to the CD40-targeting vaccines. Vaccination responses following the same administration and sampling schedule as above with anti-CD40 11B6 non-covalently linked to gp140 elicited serum anti-gp140 IgG titers that were detected only after a second vaccination (**Figure 9B**, D27 middle panel), and increased further after the third (day 21) vaccination (**Figure 9B**, D34 right panel), but the titers were substantially lower compared to the two anti-CD40 11B6-CD40L-based vaccines.

**Figure 10:**
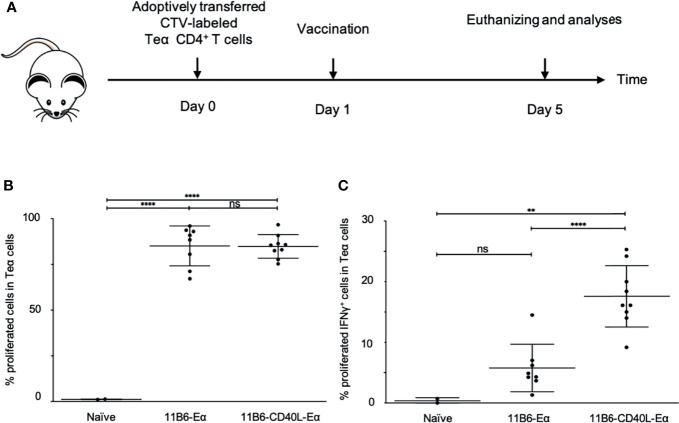
Anti-CD40 11B6-CD40L-targeted Eα antigen elicits superior proinflammatory cytokine production in Eα-specific CD4^+^ T cells in human CD40 transgenic mice. **(A)** Human CD40 homozygous transgenic mice were adoptively transferred i.v. with CTV-labeled Eα CD4^+^ T cells and 1 day later, immunized with anti-CD40 11B6-Doc complexed with Cohesin-Eα or anti-CD40 11B6-CD40L-Doc complexed with Cohesin-Eα (1 μg). Four days later cells from the skin draining lymph node were examined by flow cytometry for proliferation **(B)** and activation **(C)** of the Eα cells. To determine the activation status, some cells were stimulated with PMA/Ionomycin and then stained for intracellular IFNγ. Each dot represents a separate mouse. Data from three independent experiments were pooled. ** and **** indicate statistically significant differences as defined in *Materials and Methods*. ns indicates no significant difference.

A well-established mouse model for T cell vaccine response uses adoptively transferred CD4^+^ T cells from TCR I-Eα52-68 transgenic mice (25). We compared vaccination with anti-CD40 11B6 *versus* anti-CD40 11B6-CD40L for targeted delivery of Eα antigen for evoking proliferation and activation of these adoptively transferred cells (**Figure 10A**). In both cases, in response to a single 1 µg dose of each construct, >80% of the adopted TEα CD4^+^ T cells proliferated over the 4 days period subsequent to vaccination, indicating efficient processing and presentation of the antigen by both constructs (**Figure 10B**). However, a significantly higher proportion of the proliferating TEα CD4^+^ T cells were primed for IFNγ production by the anti-CD40 11B6-CD40L-TEα vaccine (18± *versus* 5±%, p<0.0001, **Figure 10C**).

**Figure 11:**
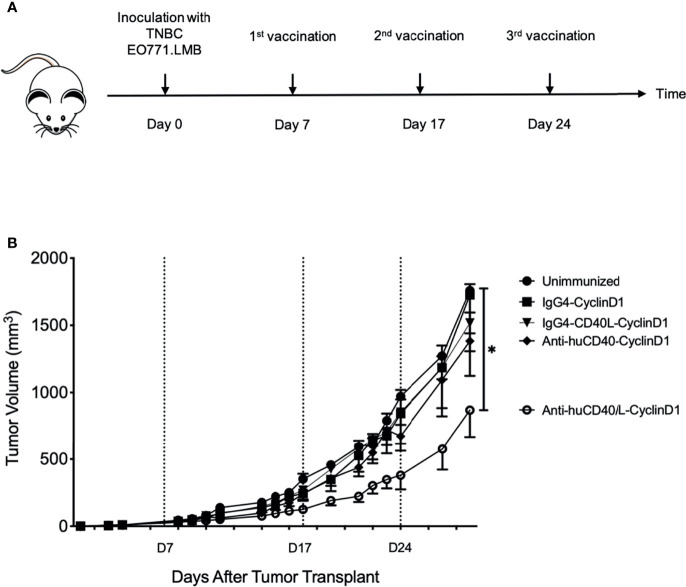
Anti-hCD40-CD40L treatment significantly reduces tumor burden in a therapeutic setting. Seven days post-inoculation with TNBC EO771.LMB tumor cells, human CD40 transgenic mice were randomized in different groups and vaccinated as indicated in **(A)** with 20 μg ant-CD40-CD40L vaccine or controls at days 7, 17, and 24. Tumor volumes were recorded by caliper measurements at the indicated time points and presented as mean ± SEM. **(B)** One representative experiment out of six is shown. Mice where the tumor did not uptake were excluded from the data. Between three and six mice were in each group. Statistics were calculated starting from D14 post tumor injection and statistical significance was P < 0.05, indicated by *.

To further explore the possible advantage in efficacy for raising cellular immunity by targeting antigen *via* anti-CD40 11B6-CD40L, we established EO771 mammary adenocarcinoma tumors [derived from a spontaneous mammary tumor in a female C57BL/6 mouse (26)] in human CD40 transgenic female mice. These tumors express Cyclin D1, a tumor-associated antigen common in breast cancers (27). At days 7, 17, and 24, mice implanted with tumor at day 0 were vaccinated with anti-CD40 11B6-CD40L-Doc, anti-CD40 11B6-Doc, IgG4-CD40L-Doc, and non-targeting IgG4-Doc non-covalently linked to human Coh-Cyclin D1 (**Figure 11A**). Only the anti-CD40 11B6-CD40L-Cyclin D1 group showed reduction in tumor growth (**Figure 11B**), indicating the low impact of non-targeted antigen, or antigen targeted to CD40 without strong CD40 activation potential, *versus* the significantly improved ability of the anti-CD40-CD40L vehicle to partially control tumor growth.

## Discussion

Discovering that linking CD40L to select agonistic anti-CD40 antibodies confers superagonist properties (13) opened an avenue to exploring potential advantages for CD40-targeting vaccines that hyperactivate CD40. Five CD40-targeting vaccines are currently in clinical development, i.e., anti-CD40-HPV16 E6/E7 (9), anti-CD40-HIV-1 Env gp140 (10), anti-CD40-HIV5pep (14, 21), and anti-CD40 fused to SARS-CoV-2 proteins (28). Although based on the agonistic anti-CD40 12E12 antibody, these antigen fusions have relatively low CD40 agonist activity. *In vivo* studies in human CD40 transgenic mice, humanized mice, and non-human primates have shown the benefit for both humoral and cellular immune responses of co-administering anti-CD40 12E12-based vaccines with poly ICLC (Hiltonol™), a TLR3-stimulating adjuvant (9–11). Early-phase clinical trials have shown that poly ICLC is safe and well tolerated (29), but it is attractive to consider potential benefits of delivery of antigen to APCs concomitant with activation of the same cells *via* a single vaccine product. The mechanism of reduced CD40 activation by anti-CD40 12E12 *via* antigen fusion is not known, but here we show that hyperactivating anti-CD40 11B6-CD40L antibody fused to all antigens we tested (i.e., HIV5pep, GNG, HPV E6/E7, Flu M1) maintains strong CD40 agonist activity. Our previous study with anti-CD40-CD40L fusion antibodies (13) revealed that this format clusters surface CD40 on DC very effectively and is internalized more rapidly, properties likely due to the tetravalent configuration that facilitates cross-linking and greatly stabilized CD40 off-rate. Here we confirmed that this decrease in off-rate was maintained in the context of fused antigen.

Tests with PBMC cultures using three different antigen adducts (Gag p24-Nef-Gag p17, HIV5pep, and Flu M1) demonstrated superiority of anti-CD40-CD40L-based targeting for eliciting expansion of antigen-specific CD8^+^ T cells, and increased efficacy at very low doses for eliciting antigen-specific CD4^+^ T cells. For example, in HIV-1^+^ donors comparing highly activating anti-CD40 11B6-CD40L *versus* non-activating anti-CD40 11B6 or anti-CD40 12E12 antibodies fused to HIV-1 antigens revealed no overall changes in the repertoire of expanded antigen-specific memory T cells. However, the highly activating anti-CD40 11B6-CD40L-HIV5pep, anti-CD40 11B6-CD40L-GNG, or anti-CD40 11B6-CD40L:Cohesin-Flu M1 vaccines were consistently more effective *in vitro* at expanding antigen-specific CD8^+^ T cells. The role of the CD40-CD40L pathway in inducing CD8^+^ T cell activation into cytotoxic T cells has been extensively studied (30–35). In particular, it has been shown that one way of activating CD8^+^ T cells during the course of an infection involves interaction between CD40L-expressing activated CD4^+^ T cells and the CD40-expressing DCs, which then feeds back to increased activation of the DCs, thus further stimulating nearby antigen-specific CD8^+^ T cells (30). In our model we are immediately inducing this DC superactivation through the combined action of CD40 binding *via* the anti-CD40 mAbs and the engagement of CD40L directly fused to the light chain of the antibodies. We have demonstrated that this platform induces a stronger DC activation compared to anti-CD40 mAbs or CD40L form acting alone (13), and this may overcome the necessity to engage help from antigen-specific CD4^+^ T cells. An alternative model to account for preferential expansion of antigen-specific CD8^+^ T cells observed *in vitro* involves the anti-CD40-CD40L vaccine serving as a surrogate for direct interaction between the CD40L-expressing CD4^+^ T cells and CD40-expressing CD8^+^ T cells, by directly binding CD40-expressing CD8^+^ T cells, playing a more direct role in their activation. However, by knowing the two antibodies do not cross compete (13), we tested an equimolar dose of anti-CD40 11B6-CD40L as a potential adjuvant added to cells cultured with anti-CD40 12E12-Flu M1, and this did not increase the cluster 1 CD8^+^ T cell response (data not shown). In some cases, there was a commensurate reduction in the extent of antigen-specific CD4^+^ T cell expansion, although this could be simply due to competition in the culture by the more aggressively expanding CD8^+^ T cells.

Importantly, by directly probing presentation of melanoma gp100 Class I peptide gp100 209-217 in the context of cell surface HLA-A2, we demonstrated that anti-CD40 11B6-CD40L targeting of this antigen resulted in significantly more sustained presentation of this antigen on MDDCs than delivery by anti-CD40 11B6 or anti-CD40 12E12 vehicles, another characteristic that can induce a stronger CD8^+^ T cell response. This suggests anti-CD40 11B6-CD40L targeting offers further improvement of this important immune parameter over what has already been documented for anti-CD40 12E12 delivery *versus* targeting to other DC receptors (8). This more sustained Class I antigen presentation may be a consequence of the more efficient internalization we observed with anti-CD40 11B6-CD40L antibody compared to anti-CD40 11B6 or anti-CD40 12E12 antibodies (13).

The observed *in vitro* increases in efficacy and potency for eliciting, respectively, antigen-specific CD8^+^ and CD4^+^ T cell responses raise the possibility that anti-CD40-CD40L-based antigen fusions may have advantages when used *in vivo*. In particular, the high agonist activity of this novel vaccine format offers promise of efficacious immune responses when used without co-administered adjuvant. The anti-human CD40 antibodies in our study do not cross-react with mouse CD40; thus, non-human primates or humanized mice are the most relevant *in vivo* models to test the efficacy of CD40-targeting vaccines based on highly activating anti-CD40-CD40L-antigens. As a prelude to such studies, we used human CD40 transgenic mice to probe possible advantages of anti-CD40 11B6-CD40L vaccines for inducing antigen-specific immune responses in the absence of adjuvant. In three adjuvant-free *in vivo* studies, we demonstrated that (i) anti-CD40 11B6-CD40L-HIV-1 Env gp140 elicited more potent CD40-targeting dependent anti-Env gp140 antibody responses than anti-CD40 11B6-HIV-1 Env gp140; (ii) anti-CD40 11B6-CD40L-Eα and anti-CD40 11B6-Eα were equally effective in driving Eα-specific CD4^+^ T cell proliferation, but the anti-CD40 11B6-CD40L-Eα vaccine elicited more potent Eα-specific CD4^+^ T cell IFNγ production; and (iii) anti-CD40 11B6-CD40L-Cyclin D1 vaccination inhibited the growth of transplanted Cyclin D1^+^ EO771 mammary adenocarcinoma tumors, while anti-CD40 11B6-Cyclin D1 and control IgG4-Cyclin D1 vaccines had no effect. These pilot studies clearly suggest potential benefits of adjuvant-free DC-targeting vaccines based on highly activating anti-CD40-CD40L vehicles, providing impetus for further *in vivo* studies.

The concept of vaccination *via* co-administrating activation of CD40 concomitantly with antigen delivery has also been approached using multimeric forms of CD40L fused to antigen, and *in vivo* mouse studies have shown promising efficacy for antibody (36, 37) and protective T cell responses (38–40).

Progression of highly DC activating anti-CD40-CD40L-based vaccines with a high potential for adjuvant-free administration towards future clinical application will require additional such extended *in vivo* studies, including dose ranging, for comparing them to the current CD40-targeting vaccine + poly IC formulations in clinically more relevant humanized mouse and non-human primate studies designed to demonstrate protective immunity or viral control. Our studies using an array of antigen settings point to likely advantages in skewing *in vivo* immunity towards cytotoxic cellular immunity, as well as overall potency at low dosage, of vaccines based on this platform technology.

## Data Availability Statement

The original contributions presented in the study are included in the article/**Supplementary Material**. Further inquiries can be directed to the corresponding author.

## Ethics Statement

The animal studies were reviewed and approved by Baylor Scott and White Research Institute and South Dallas Veteran’s Administration Institutional Care and Use Committees.

## Author Contributions

VC: Investigation, methodology, formal analysis, data curation, writing—original draft, review and editing. SZ: Conceptualization, investigation, methodology, formal analysis, writing—review and editing. MM: Investigation, methodology. MK: Investigation, methodology. AB: Investigation, methodology, writing—review and editing. ZW: Investigation, methodology. JE: Investigation, methodology. BI: Conceptualization, funding acquisition, supervision, planning, writing—review and editing. YL: Conceptualization, funding acquisition, supervision, visualization, writing—review and editing. GZ: Conceptualization, methodology, formal analysis, data curation, funding acquisition, supervision, writing—original draft. All authors contributed to the article and approved the submitted version.

## Funding

A Roche Collaborative Research Grant to the Baylor Scott & White Research Institute supported the initial development of anti-CD40 antibodies with enhanced agonist potency, and the Vaccine Research Institute *via* the ANR-10-LABX-77 grant funded the rest of the work. BI is supported by NIH R01AI146420.

## Conflict of Interest

The authors VC, SZ, YL, and GZ are inventors on patent application WO2020193718.

The remaining authors declare that the research was conducted in the absence of any commercial or financial relationships that could be construed as a potential conflict of interest.

## Publisher’s Note

All claims expressed in this article are solely those of the authors and do not necessarily represent those of their affiliated organizations, or those of the publisher, the editors and the reviewers. Any product that may be evaluated in this article, or claim that may be made by its manufacturer, is not guaranteed or endorsed by the publisher.
