# Supplementary material for: Anti-CD40 Antibody Fused to CD40 Ligand Is a Superagonist Platform for Adjuvant Intrinsic DC-Targeting Vaccines
Source: Front Immunol. 2022 Jan 13;12:786144. doi: 10.3389/fimmu.2021.786144 (PMC8792972; doi:10.3389/fimmu.2021.786144)
Supplement: Supplementary file 1 [file DataSheet_1.docx]

| Antibody (hIgG4) | **11B6-CD40L-HIV5pep^k^** | **11B6-CD40L-HIV5pep** | **Mega sCD40L** | **11B6-CD40L** |
| --- | --- | --- | --- | --- |
| EC_50_ (nM) IL-12 p40 | >0.1 ≤1 | >1 ≤10 | >10 | ≥0.1 <1 |
| EC_50_ / 11B6-CD40L | ≅1 | ≅10 | ≈100 | 1 |
| EC_50_ (nM) TNFα | >1 ≤10 | >1 ≤10 | >1 <10 | >0.01 ≤0.1 |
| EC_50_ /11B6-CD40L | ≈100 | ≅100 | ≈100 | 1 |
| EC_50_ (nM) IL-15 | >0.1 <1 | >1 <10 | >0.1 ≤1 | >0.01 ≤0.1 |
| EC_50_ / 11B6-CD40L | ≈10 | ≅100 | ≈10 | 1 |

**Supplemental Table 1.** Limits for the efficacious dose for 50% of maximum cytokine secretion (EC_50_) are given as nanomolar values (nM) based on the titration data shown in Fig. 2. For the MDDC data, the averaged maximal production values were IL-12 p40 (1463 pg/ml), TNFα (8356 pg/ml), and IL-15 (25 pg/ml). Anti-CD40 11B6-CD40L-HIV5pep carries the 5 concatenated HIV-1 antigen regions on the IgG4 H chain termini as previously described (Flamar et al., 2013) while anti-CD40 11B6-CD40L-HIV5pep^k^ has two of the five antigen regions on one H chain and three on the other H chain using knob-in-hole technology (Flamar et al., 2018; Ridgway et al., 1996).

**Supplemental Table 2.** Influenza Matrix 1 (FluM1) peptides sequences and solubility. Peptides are colored according to peptide group. Yellow=cluster 1, gray=cluster 2, green=cluster 3.


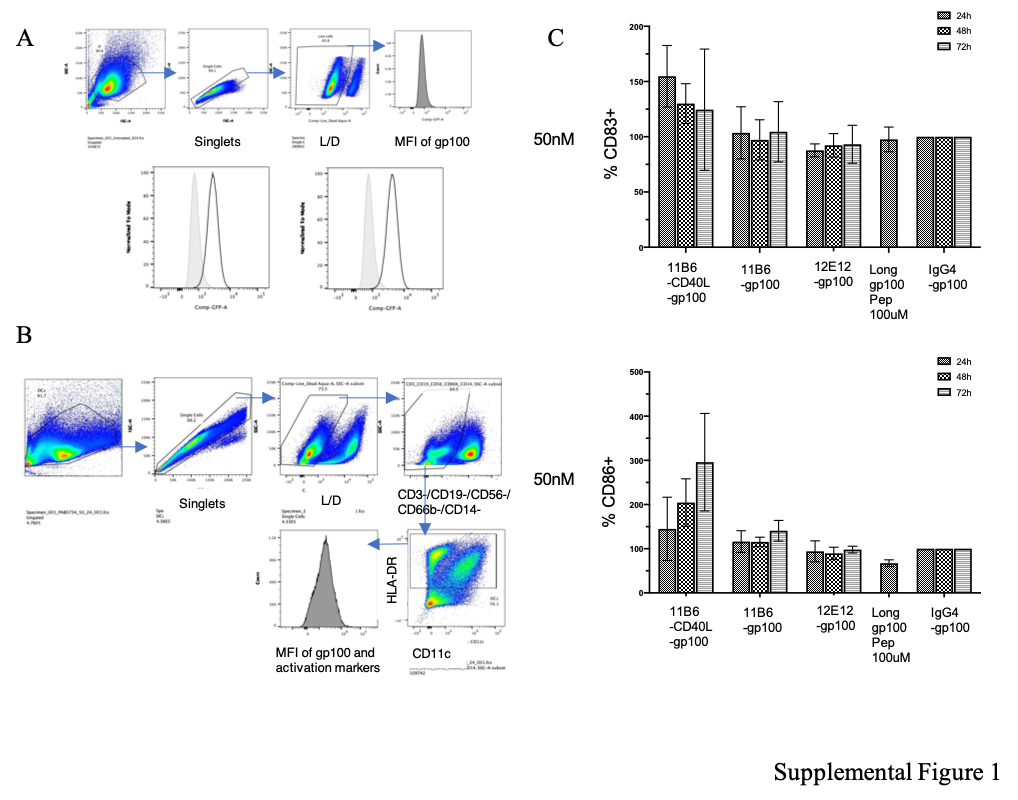


**Supplemental figure 1**. **Anti-CD40-CD40L-targeted melanoma gp100 antigen is efficiently presented on MDDC HLA-A2. (A)** JY cells can efficiently be loaded with 100 μM gp100 peptides (black line histograms), compared to the unloaded cells (grey filled histograms). **(B)** Gating strategy for FACS analyses of MDDC targeted with gp100. **(C)** Data from three independent experiments (the same as shown in Figure 8) for the total fluorescence of CD83^+^ and CD86^+^ in MDDC treated cells; cells treated with IgG4-gp100 were used as baseline set at 100% as detailed in the Fig. 8 legend.

Supplemental Figure 2

**Supplemental figure 2**. **Expansion by CD40-targeted GNG antigen of HIV-1-specific T cells in HIV-1-infected donor P3 PBMC cultures.** HIV-1^+^ donor PBMCs were cultured with a dose range of anti-CD40-GNG fusion proteins with and without a low dose of sCD40L (100 ng/ml; 6 nM) and IL-2 for 9 days, followed by stimulation with peptides for pools HIV-1 Gag p17, Nef, and Gag p24 for 6 hours, then analyzed by ICS. The data show the percentage at the end of the culture of antigen-specific (**A**) CD4^+^ and (**B**) CD8^+^ T cells producing IFNγ + TNFα in response to peptide stimulation.  This data supports the data showed in Figure 4, here replicated with a different donor (P3).

**Supplemental figure 3. CD40-targeted HIV5pep antigens with and without fused CD40L tested via *in vitro* expansion of HIV-1-specific T cells in HIV-1-infected donor PBMC cultures.**This shows ICS data for an additional **(A)** anti-CD40 12E12-HIV5 and **(B)** anti-CD40 11B6-CD40L-HIV5pep pair (using the Gen 3 antigen cassette versions). Details are the same as described for Fig. 5.
